# Mesangial Cells (MES-SV40) Cultured in High Glucose Produce IL-36α, Which Is Associated with Type 2 Diabetes Mellitus

**DOI:** 10.3390/ijms27062751

**Published:** 2026-03-18

**Authors:** María Marcela Sánchez-Torres, Cesar G. Pelcastre-Rodríguez, Fernando Gómez-Chávez, Isaí Martínez-Torres, José Martín Murrieta-Coxca, Alma Nelly Diaz-Herreros, Marcelo W. Heredia-Murillo, Juan C. Cancino-Diaz, Mario E. Cancino-Diaz

**Affiliations:** 1 Laboratorio de Inmunomicrobiología, Departamento de Microbiología, Escuela Nacional de Ciencias Biológicas del Instituto Politécnico Nacional, Mexico City 11340, Mexico; mariamarcelasat58@gmail.com; 2 Laboratorio de Inmunología Aplicada, Departamento de Inmunología, Escuela Nacional de Ciencias Biológicas del Instituto Politécnico Nacional, Mexico City 11340, Mexico; cesar_kof240@live.com.mx (C.G.P.-R.); isaimtztorres@gmail.com (I.M.-T.); nellyradios@gmail.com (A.N.D.-H.); marceloheredia80@gmail.com (M.W.H.-M.); 3 Laboratorio de Enfermedades Osteoarticulares e Inmunológicas, Sección de Estudios de Posgrado e Investigación, Escuela Nacional de Medicina y Homeopatía del Instituto Politécnico Nacional, Mexico City 07320, Mexico; fgomezch@ipn.mx; 4 Placenta Lab, Department of Obstetrics, Jena University Hospital, Am Klinikum 1, 07747 Jena, Germany; josemartin.murrietacoxca@med.uni-jena.de; 5 Centro de Investigación Científica Serendipia Asociación Civil, Tláhuac, Mexico City 13210, Mexico

**Keywords:** mesangial cells, diabetes, IL-36α, VEGF, diabetic nephropathy, angiogenesis

## Abstract

The high concentration of the inflammatory cytokine IL-36 in the serum of patients with type 2 diabetes mellitus (T2DM), along with the reduced renal damage observed in IL-36R knockout mice following ischemia–reperfusion-induced acute kidney injury, suggests a significant association between IL-36 activity and diabetic complications such as diabetic nephropathy (DN). It is also known that minor structural alterations in glomerular tissues can lead to changes in blood vessel pressure, potentially contributing to the development of DN, with inflammation acting as a triggering factor. However, further studies are needed to confirm this relationship. In this study, we observed that mesangial (MES-SV40) cells cultured under high-glucose conditions produced IL-36α in a dose-dependent manner. This cytokine production was also detected in mesangial cells from the glomerular tissues of mice with a high-calorie diet-induced T2DM, whereas healthy mice did not show such expression. In addition, we observed that mouse endothelial cells (SVECs) showed increased tubule formation in co-culture with MES-SV40 cells that had been previously exposed to 30 mmol/L glucose, as well as with the supernatant from these cells. IL-36R expression was confirmed in endothelial cells, as well as the angiogenic effect of IL-36α. Given that elevated VEGF levels have been reported in patients with DN by other authors, our results suggest that IL-36 produced by mesangial cells under high-glucose conditions may promote angiogenesis in glomerular tissues, potentially initiating the development of diabetic nephropathy.

## 1. Introduction

The IL-36 subfamily, part of the IL-1 superfamily, includes a group of inflammatory cytokines that have been relatively less studied compared to the well-characterized members of the IL-1 family. Nonetheless, IL-36 (α, β, and γ) cytokines are strongly linked to the pathological mechanisms of psoriasis, a disease that is commonly associated with diabetes [1,2,3].

Supporting this association, previous studies have reported elevated levels of IL-36α in the serum of patients with type 2 diabetes mellitus (T2DM) compared to healthy individuals. However, research into the specific role of IL-36 cytokines in T2DM-related complications remains limited [3].

One study demonstrated increased IL-36α expression in the tubular epithelial cells of diabetic mice, while another highlighted the role of IL-36α in maintaining kidney function. In that study, IL-36R knockout mice had less renal damage in the proximal tubules after ischemia–reperfusion-induced acute kidney injury, compared to wild-type controls [4].

Additionally, IL-36α production in glomerular tissues has been linked to IgA-induced mesangioproliferative glomerulonephritis, suggesting a possible role for IL-36α in diabetic nephropathy (DN). This finding aligns with previous studies, which have shown that high glucose levels can induce the expression of other inflammatory cytokines, such as TNF-α, MCP-1, IL-6, and IL-1β [5,6].

In this work we show that mesangial cells cultured under high-glucose conditions produce IL-36α that can promote endothelial activation inducing angiogenesis.

## 2. Results

### 2.1. MES-SV40 Cultured in High Glucose Produces the Release of the Inflammatory Cytokine IL-36α

Mesangial cells (MES-SV40) were cultured in glucose-free F12 medium with different concentrations of glucose (5.5, 10, 20, and 30 mmol/L) for 24 h. The mRNA levels of IL-36 cytokine family members and other inflammatory cytokines, such as IL-1β, IL-6, and TNF-α, were then analyzed.

**Figure 1:**
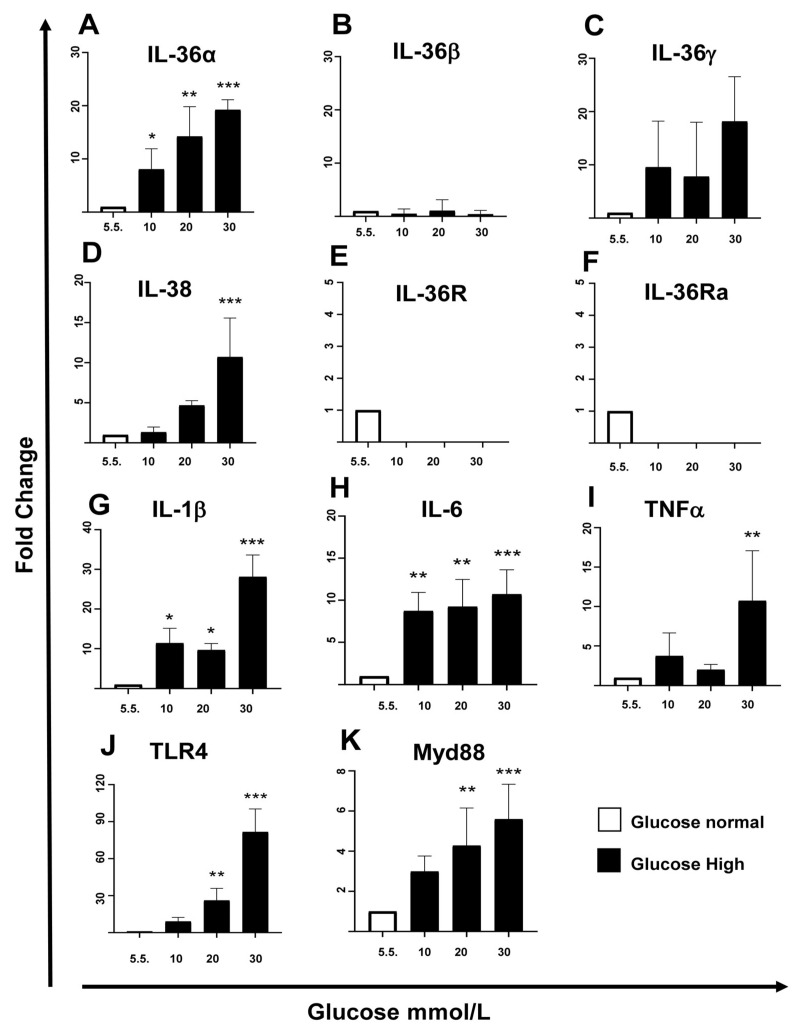
MES-SV40 cultured under high glucose conditions produces expression of mRNA of the IL-36α subfamily and other inflammatory cytokines. Cells were incubated for 24 h in glucose-free F12 medium supplemented with varying concentrations of glucose (5.5, 10, 20, and 30 mmol/L), and mRNA expression of IL-36α (**A**), IL-36β (**B**), IL-36γ (**C**), IL-38 (**D**), IL-1β (**G**), IL-6 (**H**), TNFα (**I**) cytokines and IL-36R (**E**), IL-36Ra (**F**), TLR4 (**J**), Myd88 (**K**) receptors was analyzed by RT-PCR. Three independent experiments with duplicate samples per condition were done. Statistical significance was indicated as follows: *p* < 0.05 (*, **), and *p* < 0.001 (***).

A significant dose-dependent increase in IL-36α mRNA expression was observed, with levels approximately 18 times higher in cells cultured in 30 mmol/L glucose compared to those in 5.5 mmol/L (Figure 1A). In contrast, IL-36β mRNA expression remained unchanged across all glucose concentrations tested (Figure 1B), and IL-36γ showed a non-significant trend toward increased expression (Figure 1C).

**Figure A1:**
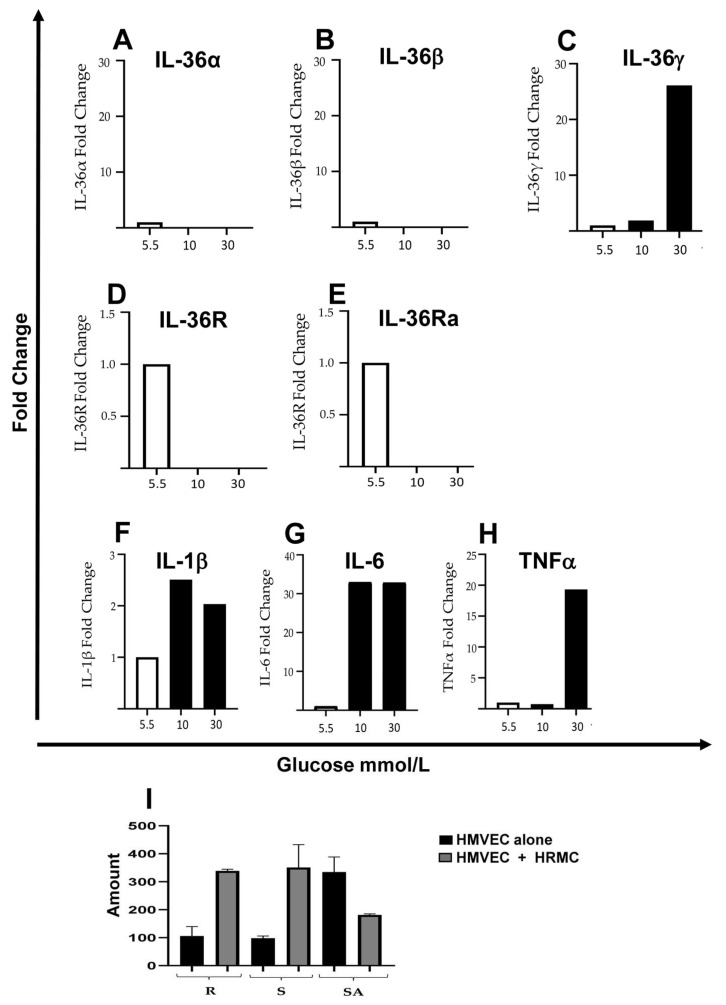
High glucose induces the expression of IL-36 subfamily and pro-inflammatory cytokines in HRMCs, promoting angiogenesis in vitro. Human renal mesangial cells (HRMCs) were cultured for 24 h in media containing increasing concentrations of glucose (5.5, 10, 20, and 30 mmol/L). mRNA expression of IL-36 subfamily cytokines and related receptors was analyzed by RT-PCR: IL-36α, IL-36β, and IL-36γ (**A**–**C**); IL-36 receptor (IL-36R) and its antagonist IL-36Ra (**D**,**E**); and classical inflammatory cytokines IL-1β, IL-6, and TNFα (**F**–**H**). The white bar corresponds to a normal glucose concentration and the black bars to high glucose concentrations. (**I**) shows the angiogenesis assay performed by co-culturing HRMCs (previously exposed to 30 mmol/L glucose) with human microvascular endothelial cells (HMVECs) in Matrigel with reduced growth factor (M-RGF). Tube formation was assessed after 4 h using bright-field microscopy and quantified with ImageJ software. The following parameters were measured: R = Branches, S = Segments and Sa = Isolated segments. All experiments were performed independently three times, with corresponding duplicates for each.

On the other hand, in a similar experiment with human renal mesangial cells (HRMCs), only IL-36γ expression was elevated at 30 mmol/L glucose, while IL-36α and IL-36β were not significantly induced (Figure A1C (Appendix A)).

Additional members of the IL-36α family, including IL-38, IL-36Ra, and IL-36R, were also examined in MES-CV40 cells. Of these, only IL-38 mRNA was significantly increased at 30 mmol/L glucose (Figure 1D). IL-38 is known to antagonize the activity of pro-inflammatory IL-36α cytokines, like IL-36Ra, which was not detected in murine mesangial cells (Figure 1F). IL-36R mRNA levels were not elevated by high glucose, although the baseline expression of the receptor cannot be ruled out (Figure 1E).

To further evaluate the expression of inflammatory cytokines, mRNA levels of IL-1β, IL-6, and TNF-α were also measured. Consistent with previous reports, high glucose levels induced the expression of these cytokines in MES-CV40 cells (Figure 1G–I). Similar results were seen in HRMCs, where IL-1β, IL-6, and TNF-α were also increased under high-glucose conditions (Figure A1F–H).

High glucose also caused a dose-dependent increase in the mRNA expression of Toll-like receptor 4 (TLR4) and its intracellular adaptor molecule MyD88 in MES-CV-4 cells (Figure 1J,K).

**Figure 2:**
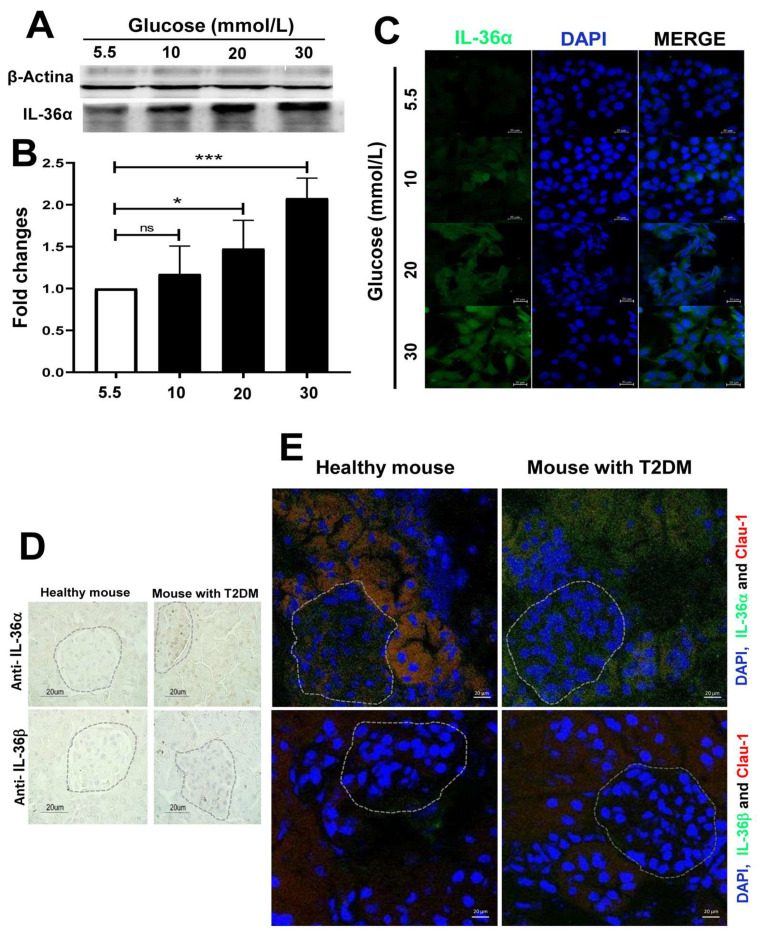
Analysis of IL-36α cytokine production in MES-SV40 cells cultured under high glucose conditions and in renal tissues from a type 2 diabetes mouse model. MES-SV40 cells were cultured for 24 h in media containing different concentrations of glucose (5.5, 10, 20, and 30 mmol/L). The expression of IL-36α was measured by Western blot analysis (**A**) and semi-quantified with image analysis software (**B**). The white bar corresponds to a normal glucose concentration and the black bars to high glucose concentrations. Additionally, IL-36α localization was examined in the same cell cultures using immunofluorescence (**C**). To induce type 2 diabetes mellitus (T2DM), C57BL/6J mice were fed either a standard diet or a high-calorie diet for 10 weeks. Renal tissues from both healthy and diabetic mice were analyzed for IL-36α and IL-36β expression with immunohistochemistry (**D**) and immunofluorescence (**E**). All images shown are representative results from eight independent experiments conducted at different times. Dashed circles indicate the glomerular regions within the renal tissue sections. Statistical significance was indicated as follows: *p* < 0.05 (*), and *p* < 0.001 (***).

Western blot analysis confirmed that IL-36α protein levels increased with higher glucose concentrations (Figure 2A,B; *p* < 0.05), aligning with the mRNA expression data shown in Figure 1. Immunofluorescence analysis further supported these findings by revealing more intense IL-36α signals in MES-CV40 cells cultured at 30 mmol/L glucose compared to those at 5.5 mmol/L (Figure 2C).

### 2.2. In a T2DM Animal Model, IL-36α Is Found to Be Overproduced in Glomerular Tissue

Given that in vitro assays showed IL-36α production in MES-SV40 cells cultured under high-glucose conditions, we aimed to investigate whether IL-36 expression was similar in vivo. For this, eight female C57BL/6J mice of 4 months old and weighing 21–24 g, which present high susceptibility to impaired insulin secretion and glucose tolerance (because they carry a deletion in the Nnt gene) [7], were fed with a high-calorie diet (HCD) for 10 weeks to induce early T2DM. Eight control mice of the same strain were kept on a standard diet (SD). Hyperglycemia was evident in HCD-fed mice from week 5 and persisted through week 10, consistent with previous findings reported by Díaz-Herreros et al. (2026) and Appiakannan et al. in 2020 [8,9].

Immunohistochemistry and immunofluorescence analyses of renal tissues showed IL-36α expression localized to the glomerular region of mice with early diabetes, which was absent in SD-fed controls (*p* < 0.05) (Figure 2D,E). IL-36α was also found outside the glomeruli in Claudin-1-positive cells, which are distal tubular epithelial cells, only in HCD-fed mice. Previous research has shown IL-36α production in distal tubules, supporting our results [10].

Consistent with the in vitro mRNA expression data from MES-VC40 cells, IL-36β was not detected in the glomerular tissues of either HCD- or SD-fed mice (Figure 2E).

### 2.3. The Supernatant of Mesangial Cell Culture in High Glucose, Which Contains IL-36α, Induces Angiogenesis In Vitro

In vitro angiogenesis assays using Matrigel demonstrated that the supernatant containing IL-36α produced by MES-SV40 cells cultured under high-glucose conditions can trigger tube formation in mouse endothelial cells (SVEC 4-10).

**Figure 3:**
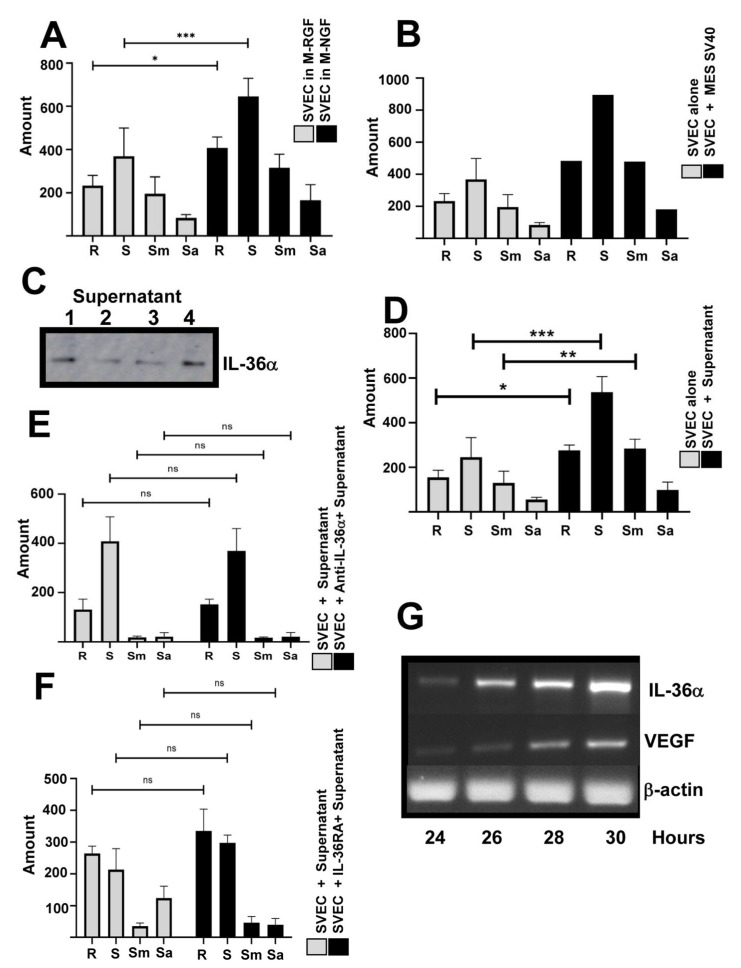
In vitro assay of angiogenesis of MES-SV40 producing IL-36α. An IL-36α detection assay was performed using endothelial cells from mice (SVECs), cultured in Matrigel with reduced growth factor (M-RGF) and normal growth factor (M-NGF) (**A**). After co-culturing SVECs with MES-SV40 (previously cultured in 30 mmol/L glucose), the cells were again cultured in Matrigel with low GF and angiogenesis was analyzed (**B**). (**C**) shows a Western blot indicating the presence of IL-36α in 4 independent supernatants from MES-SV40 cultured in 30 mmol/L glucose. The angiogenesis assay was performed with SVECs in Matrigel with low GF, in the presence of supernatants from MES-SV40 cultured in 30 mmol/L glucose (**D**). Similar experiments were conducted, but before adding the supernatants, the SVECs were incubated for 1 h with an anti-IL-36α antibody (without sodium azide) (**E**) or with the IL-36RA inhibitor (**F**). (**G**) mRNA expression of IL-36α and VEGF was analyzed by RT-PCR in MES-SV40 cultured in 30 mmol/L glucose at 24, 25, 26, and 28 h. All in vitro angiogenesis assays were analyzed at 4 h, observed under light microscopy at 60× magnification, and analyzed with ImageJ software. R = Branches, S = Segments, Sm = Meshes, and Sa = Isolated segments. The experiment was repeated three times with duplicate samples. *, ** indicates *p* < 0.05 and *** indicates *p* < 0.001.

To validate our Matrigel-based angiogenesis system, SVEC 4-10 (SVECs) were first cultured in Matrigel supplemented with either reduced growth-factors (M-RGFs) or normal growth-factors (M-NGFs) media. After 4 h of incubation in NGF-enriched Matrigel, SVECs displayed strong angiogenic activity, characterized by the formation of branches (R), segments (S), meshes (Sm), and isolated segments (Sa), indicating successful in vitro tubule formation, indicative of angiogenesis potential. In contrast, SVECs cultured in RGF Matrigel showed a significant reduction in angiogenic structures (*p* < 0.001), confirming that insufficient angiogenic stimuli impair tube formation (Figure 3A).

To investigate the role of MES-SV40 under high-glucose conditions, a co-culture system was established using MES-SV40 cells previously exposed to 30 mmol/L glucose and SVECs in RGF Matrigel. After 4 h, co-cultures showed a significant increase in angiogenic structures (R, S, Sm, IS) compared to SVECs cultured alone under the same conditions (Figure 3B). These data suggest that factors secreted by MES-SV40 cells under high-glucose conditions promote angiogenesis.

We confirmed the presence of IL-36α in 4 independent MES-SV40-conditioned media under high-glucose conditions by Western blot analysis (Figure 3C). As expected, SVECs cultured in this conditioned medium on RGF Matrigel showed increased angiogenesis at 4 h (Figure 3D). This result suggested that IL-36α promotes endothelial activation and tubule formation in endothelial cells.

Similar results were observed with human cells. Human microvascular endothelial cells (HMVECs) co-cultured with HRMCs previously exposed to 30 mmol/L glucose also exhibited increased angiogenesis (Figure A1I).

**Figure 4:**
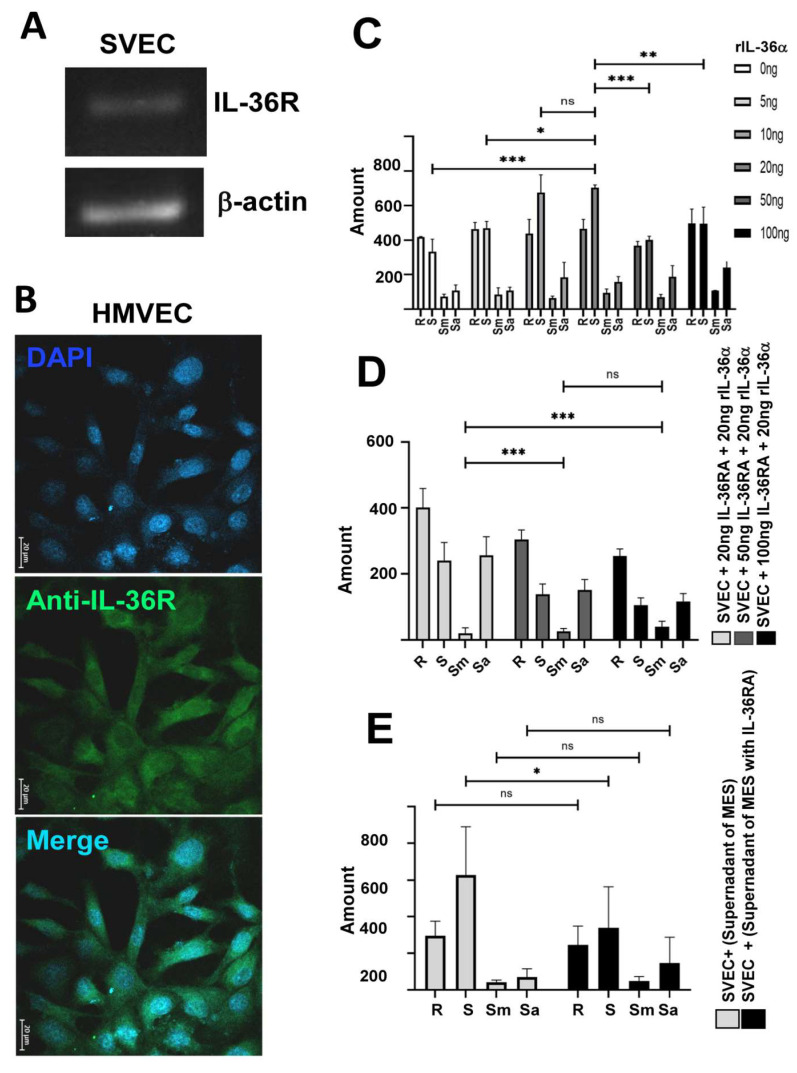
Expression analysis of IL-36R in the SVECs and HMVECs and the angiogenic effect of IL-36α in SVECs. SVECs were cultured in supplemented F12 medium, for 24 h, and RT-PCR was performed to determine mRNA expression of IL-36R (**A**). The HMVECs were cultured in F12 medium supplemented with FBS for 24 h, and FITC-Anti-IL-36R antibody was used for immunofluorescence detection (**B**). Angiogenesis assay was performed with SVECs in Matrigel with low GF in the presence of 5, 10, 20, 50 and 100 ng of recombinant IL-36α (**C**) or recombinant IL-36α combined with 20, 50, and 100 ng of IL-36RA (**D**), as well as supernatants from MES-SV40 cultured in 30 mmol/L glucose in presence of IL-36RA (**E**). *, ** indicates *p* < 0.05 and *** indicates *p* < 0.001.

To demonstrate that the IL-36α present in the conditioned medium from MES-SV40 cells cultured under high-glucose conditions is responsible for inducing angiogenesis, we performed a blocking analysis targeting both the cytokine and its receptor. When SVECs were stimulated with conditioned medium pre-treated with an anti-IL-36α antibody (Figure 3E), or with recombinant IL-36RA—a competitive inhibitor of the IL-36 receptor (Figure 3F)—angiogenesis was not fully blocked under either condition. This incomplete blockade likely reflects the fact that MES-SV40 cells cultured under high-glucose conditions also produce VEGF; indeed, expression analysis by RT-PCR revealed that these cells over-express IL-36α mRNA at 26 h, followed by a progressive increase in VEGF mRNA starting at 28 h (Figure 3G). We next investigated whether endothelial cells express the IL-36 receptor. RT-PCR analysis confirmed IL-36R expression in SVECs (Figure 4A), and immunofluorescence confirmed IL-36R expression in HMVECs (Figure 4B). Using recombinant IL-36α, we confirmed that this cytokine induced angiogenesis in SVECs in a dose-dependent manner (5, 10, and 20 ng; Figure 4C), but at high concentrations (50 and 100 ng), angiogenesis decreased. IL-36RA blocked the effect of recombinant IL-36α in SVECs (Figure 4D), confirming the functional activity of IL-36RA in these cells. To investigate a potential autocrine role of IL-36α in stimulating VEGF production by MES-SV40 cells, we applied conditioned medium from MES-SV40 cells cultured under high-glucose conditions in the presence of IL-36RA to SVECs and observed partial inhibition of angiogenesis (Figure 4E). These results suggest that IL-36α promotes VEGF production by mesangial cells (Figure 3G), which then drives angiogenesis in SVECs.

All these findings support the hypothesis that inflammatory cytokines, such as IL-36α, produced by mesangial cells under high-glucose conditions, can activate endothelial cells and promote angiogenesis in glomerular tissues, potentially contributing to the development of diabetic nephropathy in T2DM.

## 3. Discussion

Mesangial cells (MCs) are closely connected to endothelial cells within the glomerulus, and along with podocytes, they are essential for preserving the structural integrity of this kidney functional unit. Dysfunction of MCs can impair glomerular function, potentially causing nephropathy [11,12].

In the context of DN, it is well established that mesangial cell proliferation leads to the accumulation of extracellular matrix, thickening of the basement membrane, mesangial sclerosis, and interstitial fibrosis. DN is mainly characterized by diffuse, and sometimes nodular, glomerular sclerosis, which blocks glomerular capillaries and impairs kidney function [11,12,13].

Although the molecular mechanisms behind the initiation and progression of DN are not entirely clear, hyperglycemia is recognized as the main pathogenic factor. Many studies have demonstrated that high glucose conditions stimulate MC proliferation and increase extracellular matrix production. Furthermore, oxidative stress and inflammation caused by hyperglycemia are known to play a role in the development of DN. Elevated glucose levels have been shown to increase TLR4 expression and promote the production of inflammatory cytokines such as TNFα, MCP-1, IL-6, and IL-1β [14,15,16,17].

In this study, we report that high glucose levels in MES-SV40 cells induce not only the classical inflammatory cytokines IL-1β, IL-6, and TNFα, but also IL-36α and its regulatory cytokine IL-38, along with TLR4 and MyD88. IL-36α is considered a potent inflammatory cytokine, like IL-1β, IL-6, and TNFα, and is strongly linked to other inflammatory processes such as psoriasis [18,19]. In contrast, IL-38 exhibits anti-inflammatory activity by modulating IL-36α signaling, similar to the function of IL-36Ra [20,21]. The simultaneous production of IL-36α and its negative regulator IL-38 in mesangial cells under high-glucose conditions is a noteworthy finding, as concurrent expression of both cytokines has also been reported in the serum of patients with T2DM and DN, with a stronger positive correlation in DN than in T2DM. These results suggest that renal mesangial cells in patients with DN may contribute to the elevated circulating IL-36 levels observed in these patients, and that IL-38 produced by the same mesangial cells may represent a compensatory anti-inflammatory response to maintain physiological homeostasis in vital organs. [22,23]. In previous research, we demonstrated that MES-SV40 cells also produce IL-36α in response to PAMPs, such as LPS, PGN, and Poly I:C, indicating that bacterial or viral infections may further enhance IL-36α production in these cells [24].

Furthermore, we demonstrate that IL-36α is produced in vivo in MCs within the glomeruli of mice C57BL/6J with hypercaloric diet-induced T2DM; the impaired insulin secretion characteristic of these mice, resulting from a deletion in the Nnt gene, leads to chronic hyperglycemia and the meta-inflammatory state associated with diabetes [7]. These findings can be associated with increased serum IL-36α levels in patients with T2DM [3]. This evidence supports the hypothesis that similar biological processes may occur in human T2DM—and possibly in type 1 diabetes (T1DM)—and that poorly controlled diabetes could eventually lead to DN [25,26]. Other studies have documented IL-36α expression in the renal epithelial cells of the distal tubule in experimental models of kidney injury [10,22]. It is well established that DN is more prevalent in men than in women, as testosterone appears to predispose individuals to disease onset; men more commonly present with severe albuminuria, while in the women is more common observed renal chronic disease without albuminuria [27]. Although urinary albumin was not measured in our female mice with early-stage diabetes, IL-36α production was detected in the renal tissues of these animals. Our results confirm IL-36α production in these cells of mice with early diabetes, emphasizing the potential role of this cytokine in renal damage.

To investigate the potential role of IL-36α produced by MCs under high glucose conditions, we conducted in vitro angiogenesis assays. We found that factors released, including IL-36α, enhance tube formation. Similar findings have been reported in trophoblast cells that produce IL-36α, which also promote angiogenesis when co-cultured with endothelial cells in Matrigel [28]. Bridgewood et al., in 2018, also demonstrated that IL-36 induces angiogenesis in vitro [29].

These findings indicate that IL-36α may initiate abnormal angiogenesis in vivo within the glomerulus, contributing to glomerular damage in patients with uncontrolled T2DM and potentially causing DN. Several studies support this idea, emphasizing the strong link between VEGF activity and DN [30,31,32]. Elevated VEGF levels have been found in the serum and urine of patients with severe DN, and irregular blood vessel formation has been observed in glomerular tissues of individuals with T1DM and T2DM [33,34]. Furthermore, anti-VEGF therapies have shown promise in animal models, where neutralizing monoclonal antibodies or VEGF receptor tyrosine kinase inhibitors have decreased albuminuria and glomerular hypertrophy [35,36]. However, their use in humans remains a matter of controversy [37]. Notably, the absence of renal damage in IL-36R KO mice indicates a potential connection between IL-36 activity and VEGF in the molecular mechanisms of DN [4].

Although podocytes are traditionally considered the primary source of VEGF in the glomerulus, our in vitro experiments demonstrate that mesangial cells cultured under high-glucose conditions produce IL-36α followed by VEGF, and that co-culture of these cells with endothelial cells promotes angiogenesis even in the absence of podocytes. This suggests that IL-36α may directly stimulate the production of the angiogenic factor VEGF in endothelial cells or in mesangial cells themselves. To confirm that mesangial cell-derived VEGF production is at least partially mediated by IL-36α, we applied conditioned medium from MES-SV40 cells cultured under high-glucose conditions in the presence of IL-36RA to SVECs and observed partial inhibition of angiogenesis. VEGF production by mesangial cells has been previously reported, although without association with IL-36 cytokines. Pfäfflin et al. (2006) reported that mesangial cells overexpressing GLUT-1 produce elevated levels of IL-6 and VEGF via HIF-1α and AP-1 [38]. It has been reported that enhanced glucose uptake increases lactate and pyruvate in cells, which are end products of glycolysis, and are responsible for stabilizing HIF-1α and the production of VEGF [39]. However, other HIF-1α-independent pathways in VEGF production have been reported in other cells. VEGF can also be expressed through TLR4 signaling in odontoblast cells [40] and in cholangiocarcinoma cells, where VEGF production is controlled by the TLR4/NF-kB pathway [41], and by TLR4/MAP phosphorylation, as well as TLR4/Akt signaling pathways in other cells [42,43]. In hepatocellular carcinoma, VEGF production has also been shown to be controlled by the TLR4/MyD88 and STAT3/Sp1 pathways. We observed that MES/SV40 cultures under high-glucose conditions exhibited elevated mRNA expression of TLR4, MyD88, IL-36α, and VEGF. All of the above suggest that VEGF expression in MES can be mediated by both TLR4 and IL-36, as neutrophils have been reported to activate a TLR4/IL-36R crosstalk, leading to activation of the MyD88/NF-kB signaling pathway and cytokine production [44]. The MES/SV40 also produced IL-6, a cytokine associated with DN [45], which induces HIF-1α via STAT3 in inflammatory cells [46].

Taken together, our results suggest that high glucose levels induce mesangial cells to produce IL-36α, which may subsequently promote the generation of angiogenic factors in endothelial cells. This process could lead to abnormal angiogenesis and glomerular injury, characteristic features of diabetic nephropathy [30,47,48].

## 4. Materials and Methods

### 4.1. Culture and Stimulation of MES-SV40 Mesangial Cells

Mouse mesangial cells from the MES-SV40 cell line (ATCC, CRL-1927) were cultured in DMEM: F12 medium (1:2 ratio; Gibco, Waltham, MA, USA) supplemented with 5% fetal bovine serum (FBS) (Gibco, Waltham, MA, USA), at 37 °C in a humidified atmosphere containing 5% CO_2_.

For glucose stimulation assays, MES-SV40 cells were seeded in six-well plates and grown to 95% confluence in glucose-free DMEM (Gibco) supplemented with 2% FBS. Each well was treated as an independent experimental condition. Before stimulation, cells were incubated in serum-free DMEM for 1 h. Glucose concentrations of 5.5, 10, 20, and 30 mmol/L were achieved by adding glucose monohydrate directly to the medium. These concentrations represent normoglycemic (5.5 mmol/L) and hyperglycemic (10–30 mmol/L) conditions. Media were refreshed every 12 h to maintain stable glucose levels throughout the 24-h incubation period.

### 4.2. Gene Expression Analysis by RT-PCR

At the end of the incubation period, supernatants were collected and stored at −20 °C for subsequent analyses. Total RNA was extracted from cells using TRIzol^®^ reagent (Invitrogen, Waltham, MA, USA). Briefly, 500 μL of TRIzol was added to each well, and the samples were stored at −20 °C. RNA extraction was performed by adding 100 μL of chloroform, vortexing for 15 s, and centrifuging at 10,000 rpm for 15 min at 4 °C. The aqueous phase was collected, and RNA was precipitated with 500 μL of isopropanol overnight at −20 °C. The next day, samples were centrifuged at 10,000 rpm for 10 min at 4 °C. The RNA pellet was washed twice with 75% ethanol and once with 95% ethanol, each followed by centrifugation at 5000 rpm for 5 min at 4 °C. The pellet was air-dried and resuspended in 20 μL of nuclease-free water. RNA concentration and purity were assessed using a NanoDrop 2000^®^ spectrophotometer (Thermo Scientific, Waltham, MA, USA).

For reverse transcription, 2 μg of total RNA was used per sample following the manufacturer’s protocol (Invitrogen, Waltham, MA, USA). The reaction mixture included 1 μL of 10 mmol/L dNTPs, 1 μL of oligo(dT) (0.5 μg/μL), and nuclease-free water to a final volume of 12 μL. Samples were incubated at 65 °C for 5 min, followed by the addition of 4 μL of 5× First Strand Buffer and 2 μL of 0.1 M DTT. After a 2-min incubation at 37 °C, 200 U of M-MLV reverse transcriptase was added, and the reaction was carried out at 37 °C for 50 min.

PCR amplification was performed using 1 μL of cDNA, 1 μL of each primer (Table 1, 0.2 μM), and 25 μL of MyTaq™ Red Mix 2× (Meridian Bioscience, Cincinnati, OH, USA). PCR conditions were optimized based on the melting temperature of each primer pair. Amplified products were resolved on 2% agarose gels stained with GelRed (Biotium, Waltham, MA, USA). Band intensity was quantified using a ChemiDocTM Touch Imaging System (Bio-Rad, Hercules, CA, USA) equipment, and data were analyzed using the Image LabTM 6.0.1 software (Bio-Rad, Hercules, CA, USA).

Expression levels were normalized to β-actin and calculated as fold change relative to the control condition (5.5 mmol/L glucose).

### 4.3. Western Blot

Protein extracts were obtained from MES-SV40 cells after 24-h glucose stimulation. Supernatants were collected and stored at −20 °C. Cells were washed with phosphate-buffered saline (PBS, pH 7.4) and lysed using a buffer containing Cell Signaling lysis reagent and 1% protease inhibitor cocktail (SIGMA FAST, St. Louis, MO, USA). Lysates were stored at −20 °C until analysis.

Protein concentration was determined using the DC Protein Assay Kit (Bio-Rad, Hercules, CA, USA). Equal amounts of protein (50 μg) were separated by SDS-PAGE and transferred to nitrocellulose membranes (Bio-Rad). Membranes were blocked with 0.2% gelatin in PBS for 1 h at room temperature. Primary antibody against IL-36α/IL-1F6 (goat polyclonal; R&D Systems, Minneapolis, MN, USA) was diluted 1:500 in blocking buffer and incubated for 1.5 h at 37 °C with agitation. Membranes were washed four times with 0.1% PBS-Triton X-100 and incubated with HRP-conjugated rabbit anti-goat secondary antibody (Sigma-Aldrich, St. Lois, MO, USA) at 1:4000 dilution for 1 h at 37 °C. After additional washes, bands were visualized using ChemiDoc Touch imaging system (Bio-Rad, Hercules, CA, USA) and quantified with Image Lab 6.1 software. β-actin was used as a loading control, detected with HRP-conjugated anti-actin antibody (Santa Cruz Biotechnology, Dallas, TX, USA; 1:2000 dilution). Protein expression was normalized to β-actin and expressed as fold change relative to control.

### 4.4. Immunofluorescence Staining

MES-SV40 cells (1.5 × 10^6^) were cultured and exposed to glucose concentrations of 5.5, 10, 20, and 30 mmol/L for 24 h. After incubation, cells were washed with PBS and fixed with 4% paraformaldehyde (Sigma-Aldrich, St. Lois, MO, USA) for 20 min at room temperature. Permeabilization was performed using sodium dodecyl sulfate (SDS; Thermo Scientific, Waltham, MA, USA) for 5 min, followed by 0.2% Triton X-100 for an additional 5 min. Blocking was carried out with 3% bovine serum albumin (BSA) for 30 min at room temperature.

Cells were incubated with goat anti-IL-36α/IL-1F6 primary antibody (R&D Systems) at 1:100 dilution for 1.5 h at 37 °C. After washing with 0.1% PBS-Triton X-100, FITC-conjugated anti-goat IgG secondary antibody (Invitrogen, Waltham, MA, USA) was added at 1:100 dilution and incubated for 1.5 h at room temperature in the dark. Nuclei were stained with 1 μg/mL DAPI (Thermo Scientific, Waltham, MA, USA) for 20 min. After final washes, samples were mounted using VECTASHIELD^®^ (Vector Laboratories, Newark, CA, USA) antifade mounting medium and visualized using an LSM 710 NLO confocal microscope (Carl Zeiss, Jena, Germany).

### 4.5. Murine Model of Type 2 Diabetes Mellitus (T2DM)

The murine model of T2DM was established following the protocol described by Diaz-Herreros et al. 2026 and Appiakannan et al. in 2020 [8,9]. Briefly Four-month-old female C57BL/6J mice weighing between 21 and 24 g were used. The mice were randomly divided into two groups. Eight animals were included in a group that received a standard diet (SD) which was Lab Rodent Diet 5001 (Purina, Richmond, IN, USA) and water ad libitum for 10 weeks, while the other group, with eight mice, was fed a hypercaloric diet (HCD) prepared as follows: 1 kg of ground pellets (Lab Rodent Diet 5001), 8 eggs, 400 g of lard, and 260 g of wheat flour were mixed. All ingredients were homogenized, poured into molds, and frozen for storage. The composition of this diet is approximately: protein 12.63%, fat 29.16%, carbohydrates 29.67%, and fiber 5.72%. Additionally, water containing 20% sucrose was provided ad libitum. The total caloric intake for mice on the SD was approximately 362.43 kcal, while mice on the HCD consumed an average of 482.54 kcal.

All mice were housed in groups of three per cage in a room with a 12-h light/dark cycle for 10 weeks. Fasting blood glucose levels were checked weekly for 10 weeks using a glucometer Accu-Chek Instant, (Roche Diagnostics, Basel, CH, Switzerland) from a drop of peripheral blood from the mice’s tails. The mice fed with HCD in week 10 presented hyperglycemia (150.13 mg/dL ± 9.8) compared to 131.4 mg/dL ± 2.7 in the SD group. A post-challenge glycemia assay was also conducted at 10 weeks of study. The area under the curve AUC, (mg/dL min) of HCD-fed mice was significantly higher than that of SD-fed mice (*p* < 0.05). The mice HCD-fed also had an increase in serum insulin levels (30.41 pg/dL ± 11.94, *p* < 0.05) and serum triglycerides (391.2 mg/dL ± 102.5, *p* < 0.01) in comparison to mice fed with SD, which had insulin of 10.07 pg/dL ± 9.85 and triglycerides of 180.123 ± 45.79 mg/dL. The HOMA index was 1.86 ± 0.49 in mice fed HCD compared to 0.52 ± 0.07 in mice fed SD (*p* < 0.01). All this data confirmed that, in week 10, all mice fed HCD had early type 2 diabetes mellitus. Then, at this point, all the animals in the study were euthanized with a lethal dose of pentobarbital via intraperitoneal injection. The procedure and dosage were performed according to the guidelines established by the Mexican Official Standard for the care and use of laboratory animals (NOM-062-ZOO-1999). Kidneys were collected, fixed in 4% formaldehyde, and embedded in paraffin for histological analysis. All procedures, including euthanasia and disposal of biological material, were carried out in accordance with the bioethical guidelines set by the Research Ethics Committee of the ENCB-IPN (approval code: No. Z00-001-2021 and approval date: 29 June 2021).

### 4.6. Tissue Staining by Immunohistochemistry and Immunofluorescence

To evaluate IL-36α and IL-36β expression in mouse renal tissue, paraffin-embedded kidney sections (5 μm thick) were prepared using a standard microtome. Sections were mounted on electrostatically charged slides, deparaffinized by heating, and washed twice with xylene. Tissue rehydration was performed through a graded ethanol series (96%, 80%, and 70%), followed by three rinses in distilled water.

Antigen retrieval was carried out by placing the slides in a pressure cooker for 18 min using 1× citrate buffer. After cooling, endogenous peroxidase activity was quenched using a 3% hydrogen peroxide/methanol solution (1:8 ratio). For immunohistochemistry (IHC), endogenous avidin and biotin were blocked using commercial reagents (Zymed-Invitrogen, Waltham, MA, USA). Non-specific binding sites were blocked with 5% non-immune rabbit serum (Sigma-Aldrich) for 20 min at 37 °C in a humidified chamber. For immunofluorescence (IF), blocking was performed using 5% bovine serum albumin (BSA) for 30 min at room temperature.

Primary antibodies against IL-36α and IL-36β (goat polyclonal anti-IL-36α/IL-1F6; R&D Systems, Minneapolis, MN, USA) were applied at appropriate dilutions (1:100 for IF; 70–100 μL per slide for IHC) and incubated for 1.5 h at 37 °C (IF) or 20 min at 37 °C (IHC). After incubation, the slides were washed four times with PBS containing 0.1% Triton X-100 (IF) or PBS alone (IHC), with each wash lasting 5 min and performed with gentle agitation.

For IHC, biotinylated secondary antibodies were applied using the Histostain^®^ detection system (Invitrogen, Waltham, MA, USA), followed by incubation with streptavidin-peroxidase complex. Chromogenic detection was performed using diaminobenzidine (Biocare, Pacheco, CA, USA), which was prepared by mixing one drop of chromogen with 1 mL of reaction buffer. Slides were incubated for 50–60 s under light microscopy until color development was observed, then rinsed in distilled water. Counterstaining was performed with Meyer’s hematoxylin (50 μL per section for 1 min), followed by rinsing in distilled water and PBS. Slides were mounted with coverslips and analyzed under conventional light microscopy.

For IF, the secondary antibody (FITC-conjugated anti-goat IgG; Invitrogen, Waltham, MA, USA) was applied at a 1:100 dilution and incubated for 1.5 h at room temperature in the dark. After four washes with PBS containing 0.1% Triton X-100, nuclei were counterstained with 1 μg/mL DAPI (Thermo Scientific, Waltham, MA, USA) for 20 min. Final washes were performed using PBS-Triton X-100, and the slides were mounted with VECTASHIELD antifade mounting medium (Vector Laboratories, Newark, CA, USA). Fluorescence imaging was conducted using an LSM 710 NLO confocal microscope (Carl Zeiss, Jena, Germany).

### 4.7. In Vitro Angiogenesis Assays

Angiogenesis assays were conducted using mouse endothelial cells (SVEC 4-10; ATCC, CRL-2181) cultured in DMEM supplemented with 10% FBS (Gibco) at 37 °C in a 5% CO_2_ atmosphere. All media contained 1% penicillin-streptomycin (Gibco), and assays were performed according to the protocol described by Wiest, et al. (2014) [49].

Assays were performed in 96-well plates with 30 μL of Matrigel^®^ (Corning, NY, USA) per well. Matrigel with high or low concentrations of angiogenic factors (high FG or low FG) was allowed to solidify at 37 °C for 30 min. Subsequently, 18 × 10^3^ SVEC 4-10 cells were seeded per well in 60 μL of DMEM with 10% FBS and incubated for 6 h. Angiogenesis was evaluated at 2, 4, and 6 h, with 4 h identified as optimal time points. After 5 h, cell–cell interactions began to deteriorate. Angiogenesis assay using Matrigel low FG and recombinant IL-36α alone or with IL-36RA, as well as an anti-IL-36α antibody, was also developed.

Quantitative analysis of angiogenic parameters—including number of branches (R), segments (S), master segments (Sm), and isolated segments (Sa)—was performed using the Angiogenesis Analyzer plugin in ImageJ, v1.54p.

#### 4.7.1. Supernatant-Based Angiogenesis Assays

Supernatants from MES-SV40 cells cultured under high-glucose conditions (30 mmol/L), where IL-36α production was highest, were used as media for angiogenesis assays. Supernatants from MES-SV40 cultured under high glucose and in the presence of IL-36RA (to block the autocrine effect of IL-36α and not produce VEGF) were also obtained. Supernatants were centrifuged at 10,000 rpm for 5 min to remove debris. SVEC 4-10 cells (18 × 10^3^ per well) were seeded in Matrigel (low FG) and incubated with MES SV40 supernatants with or without IL-36RA. Angiogenesis was assessed at 4 h and analyzed using ImageJ.

#### 4.7.2. Co-Culture Angiogenesis Assays

Co-culture assays were performed to evaluate capillary network formation and cell–cell interactions between MES SV40 and SVEC 4-10 cells, based on the methodology described elsewhere [50]. In each well, 90,000 SVEC 4-10 cells and 90,000 MES SV40 cells were seeded in Matrigel (high FG or low FG) with 60 μL of DMEM containing 10% FBS. Angiogenesis was assessed at 2 and 4 h and quantified using ImageJ.

### 4.8. Statistical Analysis

All in vitro experiments were performed in triplicate. Data are presented as mean ± standard deviation (SD). Statistical analysis was conducted using one-way ANOVA followed by Dunnett’s post hoc test for multiple comparisons. Analyses were performed using GraphPad Prism v8.0.2. A *p*-value < 0.05 was considered statistically significant, as indicated in each figure.

## 5. Conclusions

The main contribution of our work highlights that high glucose levels induce IL-36α expression in mesangial cells both in vitro and in vivo, contributing to a pro-inflammatory environment in the glomerulus that may play a crucial role in the development of DN. The detection of IL-36α in renal tissues of diabetic mice, along with its ability to promote angiogenesis independently of podocytes, suggests that IL-36α could drive pathological vascular remodeling and glomerular injury. These findings emphasize IL-36α as a potential molecular link between hyperglycemia-induced inflammation and abnormal angiogenesis, offering new insights into the mechanisms of diabetic kidney damage and suggesting a possible therapeutic target.

## Figures and Tables

**Table 1:** Primer pairs used for gene expression analysis.

Primer (Tm 60 °C)	Forward	Reverse
β-actina	ATGTGGATCAGCAAGCAGGA	AAAGGGTGTAAAACGCAGCTC
IL36α	GCAAACAGTTCCAGTCACTAT	GGGTGTCTTTGATTGCTTCTT
IL36β	TGCATGGATCCTCACAATC	GGCTATAAACCAGCCAGGATA
IL36γ	CACAGAGTAACCCCAGTCAG	TTGGTCCTGCTTACCTTTCA
IL36Ra	AAGCCAGTGCCTGTCATGT	GACAGGCTGATCGGCTTCAG
IL36R	GTCCTTCAGACCTCTCCTG	CGGTTAGGTTCACAGCTATTT
IL38	ACAAACCACCCGTTTCACCT	CAGTATGGGTGGAGGGTTCA
IL1β	GCCACCTTTTGACAGTGATGA	GTGCTGCTGCGAGATTTGA
IL6	CCTCTCTGCAAGAGACTTCCAT	AGCCTCCGACTTGTGAAGTGGT
TNFα	GCGGGGCAGCCTTGTCCCTT	GCCCACGTCGTAGCAAACC
TLR4	TCCCTCCTGTGACAGTGCTA	CAATTGGCAACCAACTGGCTC
MyD88	CCAGGCATCCAACAAACTGC	CATACCCTTGGTCGCGCTTA
VEGF	CTTGCAGATGTGACAAGCCAA	AGCAGCAGATATAAGAAAATGGCG

## Data Availability

The original contributions presented in this study are included in the article. Further inquiries can be directed to the corresponding authors.

## Abbreviations

The following abbreviations are used in this manuscript:

MD	diabetes mellitus
NF	Nephropathic Diabetic
MES-SV-40	Mesangial cells mouse
HRMS	Mesangial cells
